# Trends of Diagnosis, Disease Course, and Treatment of Atopic Dermatitis 2012–2021: Real-World Data from a Large Healthcare Provider

**DOI:** 10.3390/jcm13010281

**Published:** 2024-01-04

**Authors:** Clara Weil, Roni Adiri, Gabriel Chodick, Merril Gersten, Eran Cohen Barak

**Affiliations:** 1 Maccabi Institute for Research and Innovation, Maccabi Healthcare Services, Tel Aviv 68125, Israel; 2 Pfizer Pharmaceuticals Israel Ltd., Herzliya 4672509, Israel; 3 School of Public Health, Faculty of Medicine, Tel Aviv University, Ramat Aviv 6997801, Israel; 4 Department of Dermatology, Emek Medical Center, Afula 18101, Israel; 5 Bruce and Ruth Rappaport Faculty of Medicine, Technion–Israel Institute of Technology, Haifa 3200003, Israel

**Keywords:** atopic dermatitis, incidence, real-world data

## Abstract

In the last decade, new treatments for atopic dermatitis (AD) have emerged. We aimed to describe trends of the diagnosis, disease course, and treatment of AD over a decade (2012–2021) using data from Maccabi Healthcare Services (a 2.7-million-member healthcare provider in Israel). The AD prevalence was stable (4.0% on 31 December 2021 vs. 4.3% on 31 December 2012). The annual AD incidence was also stable (5.8/1000 in 2012 and 5.7/1000 in 2021). AD-related treatment use was highest in the first year post-diagnosis, and it included, among children (*n* = 87,414) vs. adults (*n* = 36,865), low-potency topical corticosteroids (TCS) (41.8% vs. 27.1%), mid-potency TCS (30.1% vs. 28.1%), high-potency TCS (34.9% vs. 60.3%), topical calcineurin inhibitor (10.8% vs. 10.1%), phosphodiesterase-4-inhibitor (0.3% vs. 0.7% overall; approved in 2019), phototherapy (0.1% vs. 2.3%), and systemic/biologic treatments (13.0% vs. 13.3%). Among children diagnosed in 2012 and followed through to 2021 (*n* = 5248), 21.5% had ≥1 AD diagnosis/treatment 10 years later (among 3223 adults: 38.3%). We conclude that the incidence and prevalence rates of AD were comparable to those in similar database studies and remained relatively stable over the past decade. The results underscore the burden of medication use among children and adults, particularly in the first year after AD diagnosis, and the low rate of AD diagnosis among patients originally diagnosed as children 10 years earlier.

## 1. Introduction

Atopic dermatitis (AD) is a common chronic inflammatory skin disease. In the last decade, new topical and systemic treatments have emerged, potentially affecting disease awareness, therapeutic approach, and disease course.

The epidemiology of AD varies across geographical regions. While in the Western European region, the age-standardized burden of AD is among the highest in the world, Central Europe and Eastern Europe are among the five regions of the world with the lowest rates [1]. In the last two decades, there have been remarkable advances in revealing the mechanisms underlying AD, diagnosing AD, and developing therapeutic modalities.

The available approved therapies to alleviate the symptoms of AD were only topical corticosteroids (TCS) and topical calcineurin inhibitors (TCI) until December 2016. Despite their efficacy, long-term use is restricted due to cutaneous and systemic adverse reactions (TCS) and application site reactions (TCI). In December 2016, crisaborole 2% ointment, a topical phosphodiesterase-4 inhibitor (PDE4i), was approved by the US FDA for the treatment of mild-to-moderate AD. Crisaborole was approved and became available in Israel in 2019 and was reimbursed in 2021. In March 2017, dupilumab, a human monoclonal antibody targeting IL-4 and IL-13 signaling, was approved for the treatment of moderate-to-severe AD and was approved and reimbursed in Israel in January 2019. More recently (since 2022), Janus kinase inhibitors, such as abrocitinib, baricitinib, and upadacitinib, have been approved globally and in Israel for moderate to severe AD.

Appreciating the temporal trends of AD across sex/age groups and patterns of therapeutic intervention should help to better understand the AD disease burden and to plan healthcare resource allocation that will meet patients’ needs. This study aimed to describe trends of the diagnosis, disease course, and treatment of atopic dermatitis over a decade between 2012 and 2021, using a large healthcare database in Israel.

## 2. Materials and Methods

### 2.1. Study Design and Data Source

The data sources for this retrospective cohort study were the central databases of Maccabi Healthcare Services (MHS), a 2.7-million-member, state-mandated, nonprofit health provider in Israel, representing one-quarter of the population of Israel. By the National Health Insurance Law of 1994, all Israeli residents must freely choose one of the four national health providers. Membership is free and the providers are prohibited from denying membership. The MHS data include detailed demographic data, anthropometric measurements, outpatient and inpatient diagnoses, dispensed and prescribed medications, and comprehensive laboratory data from a single laboratory. MHS uses the International Classification of Diseases, Ninth Revision, Clinical Modification (ICD-9-CM) coding system and an additional coding system that was developed within MHS to improve diagnosis granularity. All medications are coded using the World Health Organization’s Anatomical Therapeutic Chemical (ATC) classifications. Procedures are classified using the Current Procedural Terminology (CPT).

### 2.2. Study Population

The identification period for AD in the database was 1998–2021. AD was defined according to any (at least one) of the following AD diagnosis criteria:

1. At least one AD diagnosis (Dx; ICD-9 691.8) from a specialist (dermatology or immunology/allergy);
2. At least one AD Dx from hospital or medical approval records or ‘active diagnosis’;
3. At least two AD Dx from a primary care physician (PCP; pediatrician or general practitioner (GP));
4. At least one AD Dx (any physician) and purchased topical calcineurin inhibitors.

Patients who met the AD definition above were eligible for inclusion in the AD prevalence population (2021) and/or the AD incidence cohort (2012–2021) defined below.

The AD prevalence population included all MHS members enrolled on 31 December 2021, with at least 12 months’ continuous health plan enrollment, who met the AD diagnosis criteria (1998–2021), with ≥1 AD diagnosis in the previous 5 years (i.e., in 2017–2021).

The study population for the main longitudinal analyses included newly diagnosed (incident) AD patients in 2012–2021, who were continuously enrolled in MHS for at least 12 months prior to their earliest AD diagnosis (‘AD diagnosis date’) and received an AD diagnosis by 31 December 2021.

### 2.3. Study Variables and Definitions

The following baseline data were obtained to describe patient characteristics at AD diagnosis. Sociodemographic characteristics included age, sex, and socioeconomic status (SES) linked to neighborhood of residence. SES was determined as a score ranked from 1 (lowest) to 10 based on an individual’s residence location, at the neighborhood level [2,3]. SES was categorized into low (1–4), medium (5–6), and high (7–10).

Clinical characteristics included BMI, smoking status (ever, never), and the presence of comorbidities. BMI was classified as underweight, normal, overweight, or obese, using BMI kg/m^2^ cutoffs of <18.5, 18.5–24.9, 25–29.9, and ≥30.0, respectively. Comorbidities were identified using existing MHS disease registries for chronic diseases (since 1998) [4,5,6,7], where available, or by using ICD-9-CM diagnosis codes (for the past 12 months).

For the first AD diagnosis, the physician specialty was described and grouped into dermatology, allergy/immunology, primary care physician (PCP; including pediatricians and family physicians), and other.

AD-related treatment use was described longitudinally starting 12 months prior to AD diagnosis, through to the end of the study period. We collected information on topical AD-related therapies—calcineurin inhibitors (TCI; pimecrolimus, tacrolimus) and corticosteroids (TCS)—as well as the following systemic therapies: immunosuppressants (SI; including 2.5 mg methotrexate, azathioprine, cyclosporine, mycophenolate mofetil, and systemic corticosteroids), PDE4i (crisaborole, available since 2019), and biologics (dupilumab). TCS potency definitions were based on the seven levels used in local clinical guidelines and categorized into low (VI–VII), mid (IV–V), or high (I–III) potency. Phototherapy was captured using CPT codes.

The baseline prevalence rates of asthma (ICD-9 493.xx) and allergic rhinitis (477.xx) were based on diagnosis given by a relevant specialist, hospital, or MHS Medication Approval Committee, or at least two diagnoses from a PCP. The baseline status of asthma and allergic rhinitis was described both ever (since 1998) and for the 5 years prior to the index date (i.e., a subset of ‘ever’ with a recent diagnosis code prior to the AD diagnosis date).

Data on healthcare resource utilization (HCRU) up to 12 months prior to AD diagnosis were obtained, including the frequency of visits to PCPs and specialists (dermatology, allergy/immunology) and hospitalization (admissions for a duration of at least 1 night, for any cause).

Among the prevalent AD patients, AD disease severity was estimated using dispensed treatments up to 5 years prior to the point prevalence assessment date (sensitivity analysis: 12 months); moderate-to-severe AD was defined by at least 1 dispensed SI or biologic, or phototherapy, with the remaining patients defined as mild AD.

### 2.4. Statistical Analysis

Descriptive statistics are reported. Numbers and percentages are provided for dichotomous and categorical variables. Continuous variables were inspected for normality (Kolmogorov–Smirnov test) and summarized accordingly as the mean and standard deviation (SD) or median and inter-quartile range (IQR). Missing data are presented as a separate category (e.g., BMI category = Missing). Longitudinal treatment patterns are described by 12-month period (i.e., at least 1 dispensed prescription per year) starting 12 months prior to AD diagnosis, through to the end of the study period; the denominator included all patients enrolled at the mid-point of a given year.

All analyses were performed using IBM SPSS Statistics version 28 (IBM Corp., Armonk, NY, USA) and R version 4.0.2 (R Foundation for Statistical Computing, Vienna, Austria).

## 3. Results

### 3.1. Trends over Time: Prevalence of AD

The prevalence of AD remained relatively stable over time during the study period. In all, 91451, 95835, and 99263 prevalent AD patients were identified at the end of years 2012, 2016, and 2021, respectively. The corresponding prevalence rates in MHS were 4.3% (95% CI: 4.3–4.4%), 4.2% (4.2–4.3%), and 4.0% (95% CI: 4.0–4.0%), respectively. In each of the years, the prevalence was observed to peak in the 5- to 9-year-old age group. Recent use of treatments suggestive of moderate-to-severe AD was also fairly consistent across years 2012 (8.4%), 2016 (8.4%), and 2021 (9.7%); we therefore focused on the most recent 2021 prevalence data to explore in detail (see also Section 3.4).

**Figure 1:**
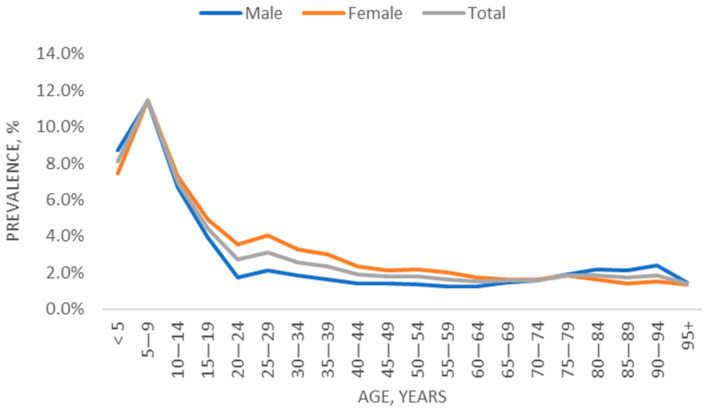
Age- and sex-specific prevalence of AD (31 December 2021).

For patients enrolled at the end of the study period (31 December 2021; patient selection described in Supplementary Figure S1), age-specific prevalence rates are depicted in Figure 1. In age groups <12, 12–17, and 18+ years, the AD prevalence rates were 9.4%, 5.7%, and 2.2%, respectively.

### 3.2. Trends over Time: Incidence of AD

**Figure 2:**
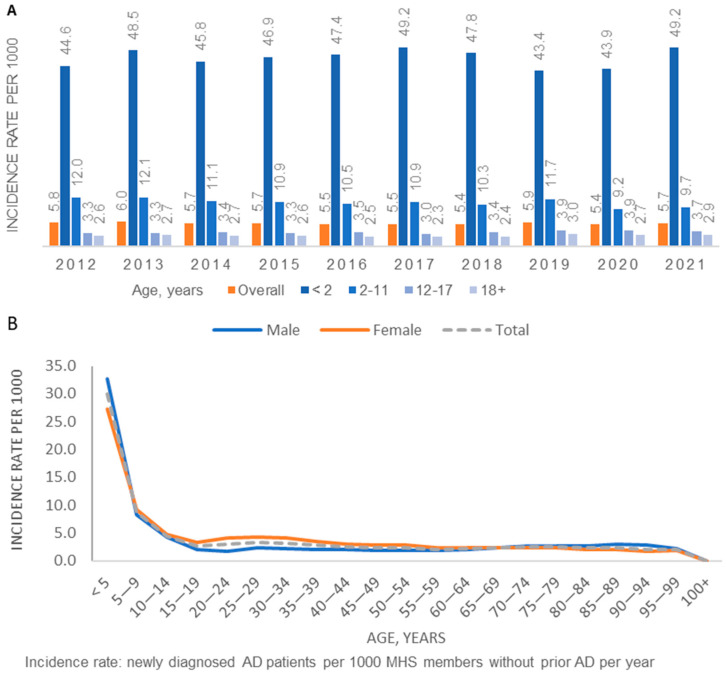
Age-specific annual incidence of AD (2012–2021): (**A**) by calendar year; (**B**) Overall and by sex (average during the study period).

A total of 124,279 incident patients were identified in 2012–2021 (Supplementary Figure S2). The average annual incidence in 2012–2021 was 5.6 per 1000 population in MHS. The annual incidence by age group and overall remained stable over the time period 2021–2021 (Figure 2a). The age-specific incidence was highest among children aged 5 and younger (Figure 2b). Compared to females, males had somewhat higher age-specific incidence in the <5-year age group and slightly lower rates in the 15–35-year age group.

### 3.3. Patient Characteristics at AD Diagnosis

Among the incident AD patients (*n* = 124,279), the median age was 4.4; IQR = 1.1–26.0 years (Table 1). Male sex was predominant (53.2%) among patients diagnosed with AD at age <12 years, while female sex accounted for the majority of patients diagnosed at an older age. Low SES accounted for 19.4% and 18.0% of patients diagnosed with AD at age <12 years and 12–18 years, respectively, compared to 15.6% of patients diagnosed in adulthood. Approximately 10% of patients in all age groups had a diagnosis of asthma in the prior 5 years. The prevalence rates of allergic rhinitis, anemia, diabetes, hypertension, CVD, and depression were highest among patients diagnosed with AD in adulthood. The ADHD prevalence was highest (18.7%) among patients diagnosed with AD in adolescence.

### 3.4. AD Disease Severity and Characteristics of Prevalent Patients (2021)

Among the prevalent patients with AD enrolled on 31 December 2021, most patients had mild AD: recent use of treatments suggestive of moderate-to-severe AD was recorded among 9.7% overall and among 12.8% of adults. Among prevalent AD patients aged <12 years old, patients who used treatments suggestive of moderate-to-severe AD had a somewhat lower SES (low SES 21.0%; high SES 49.9%) compared to patients defined as mild AD (low SES 18.2%; high SES 53.5%); a similar trend was also seen for patients aged ≥18 years. Comorbidities, notably asthma and allergic rhinitis, were more prevalent among patients defined as having moderate-to-severe AD, across all age groups (Supplementary Table S1).

### 3.5. Longitudinal Treatment Patterns of Incident AD Patients (2012–2021)

**Figure 3:**
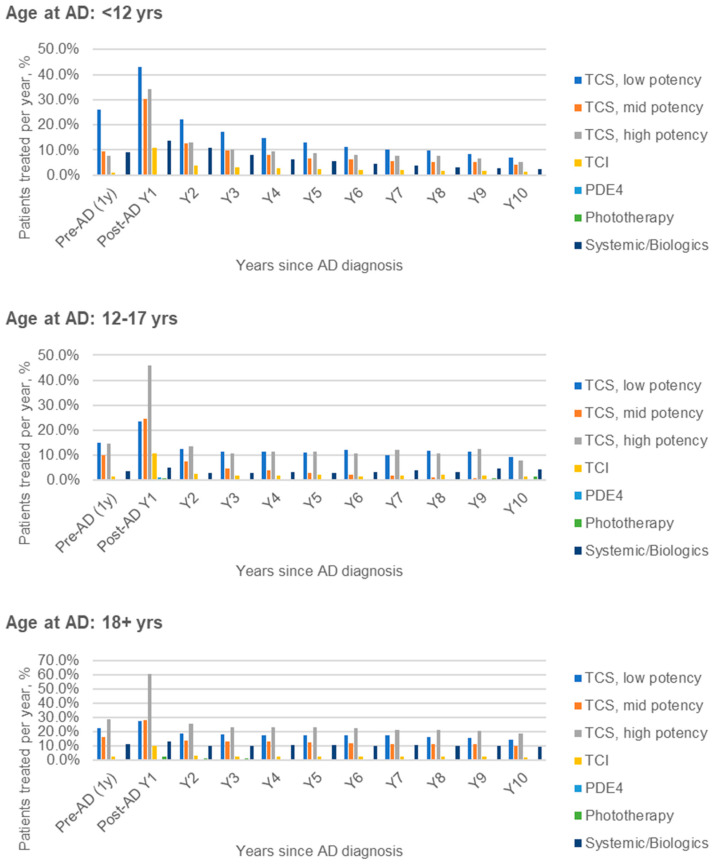
Dispensed AD-related medication over time, relative to AD diagnosis, by age at AD diagnosis. Note: treatment defined as at least 1 dispensed prescription per year. The denominator included all patients enrolled at the mid-point of a given year.

The longitudinal treatment patterns for incident AD patients diagnosed in 2012–2021 are described in Figure 3. In all age groups, some baseline use of treatments such as TCS was observed in the year prior to AD diagnosis, and all AD-related treatment use was highest in the first year following AD diagnosis. In the first year after AD diagnosis, treatment use among children (aged <18 years, *n* = 87,414) vs. adults (*n* = 36,865) was similar, except for relatively greater use of low-potency TCS and lesser use of high-potency TCS in children, and lower usage of PDE4i and phototherapy: low-potency TCS (41.8% vs. 27.1%), mid-potency TCS (30.1% vs. 28.1%), high-potency TCS (34.9% vs. 60.3%), topical calcineurin inhibitors (TCI; 10.8% vs. 10.1%), phototherapy (0.1% vs. 2.3%), systemic/biologic treatments (13.0% vs. 13.3%). The corresponding results for PDE4i use within 1 year post-diagnosis in the overall incident cohort (including years prior to PDE4i approval) were 0.3% vs. 0.7%.

### 3.6. Long-Term Patterns of AD Diagnosis and Treatment after 10 Years

To better understand long-term longitudinal patterns of AD diagnosis and treatment, a sub-analysis was performed among 11,104 patients who were newly diagnosed with AD between 1 January 2012 and 31 December 2012 and were continuously enrolled in MHS during the study period 2012–2021 (Supplementary Table S2). Overall, 5.3% of patients had a diagnosis code for AD in the year 2021, 10 years after their initial diagnosis (among children aged <12 years: 6.7%). Taking into account AD diagnosis codes and/or dispensed AD-related treatments in the year 2021, 26.1% of patients newly diagnosed in 2012 had an indication of AD in their electronic health records 10 years later; this result was highest among patients who were diagnosed in adulthood (36.2%). In a sensitivity analysis among (*n* = 5248) children (age <18 years) newly diagnosed and treated in 2012 and continuously enrolled through to 2021, 21.5% had ≥1 AD diagnosis and/or treatment documented 10 years later. The corresponding result among adults (*n* = 3223) was 38.3%.

## 4. Discussion

Using electronic healthcare data from a large healthcare provider, this study provides real-world data characterizing the epidemiology of AD over a decade. The annual incidence and prevalence of AD by age group and overall remained stable between 2012 and 2021, and the average annual incidence for this time period was 6.7 per 1000. The AD prevalence on 31 December 2021 was 4.0% overall and 9.4%, 5.7%, and 2.2% for age groups <12, 12–17, and 18+ years, respectively, with prevalence observed to peak in the 5- to 9-year-old age group. Interestingly, prevalent AD patients included a majority of males in the <12 age group and a majority of females in the 12–17 and 18+ age groups.

The reported incidence and prevalence rates are comparable to those reported in other similar database studies (including prevalence data from Israel [8,9] and other countries [1,10] and incidence data from estimates from Denmark, Sweden [11], and Norway [12]). The median reported prevalence of AD using other studies based only on routinely collected electronic healthcare data was reported to be 4.9% [10]. The prevalence rates in our analysis are also in line with recent estimates of the global prevalence of AD among adults (2.0%; 95% CI 1.4–2.6) and children (4.0%; 95% CI 2.8–5.3) [13]. Our results are consistent with evidence that AD is most commonly diagnosed in early childhood [14]. The differences in incidence and prevalence rates by sex in pediatric and adult populations are in line with evidence from other studies [15,16].

In all age groups, some baseline use of treatments such as TCS was observed in the year prior to AD diagnosis, and all AD-related treatment use was highest in the first year following AD diagnosis (Figure 3). In the first year after AD diagnosis, treatment use among children aged <18 years was similar to that among adults, except for relatively greater use of low-potency TCS, lesser use of high-potency TCS, and slightly lower use of PDE4i (crisaborole first became available in Israel in 2019 and was not approved for reimbursement until 2021) and phototherapy in children. Patients with recent use of treatments suggestive of moderate-to-severe AD accounted for 9.7% of prevalent AD patients in 2021 (among adults: 12.8%).

Data were presented summarizing the characteristics and comorbidities of prevalent patients according to age at diagnosis and severity of diagnosis. The burden of comorbidities varied by age group; among prevalent patients on 31 December 2021, asthma diagnosis in the prior 5 years was most frequently observed in the <12-year age group, ADHD was most prevalent in the 12–17 age group, and depression prevalence increased across age groups, as did age-related comorbidities such as diabetes, hypertension, and CVD. Comorbidities, notably asthma and allergic rhinitis, were more prevalent among patients defined as having moderate-to-severe AD, across all age groups. Overall, AD patients had a high burden of comorbidities, and in addition to patient distress, these may also contribute significantly to the economic burden of AD [17,18,19].

Residence in higher-SES areas was common among AD patients and was slightly higher among those who received treatments associated with milder AD, which may reflect improved access to care and increased awareness among patients with higher SES [20]. Asthma and allergic rhinitis were prevalent among both prevalent and newly diagnosed AD patients, which suggests that these atopic conditions do not necessarily develop in sequential progression after AD diagnosis as defined by the atopic march model [21].

Data on the longitudinal treatment patterns for incident patients were consistent with treatment guidelines [22], with frequent use of TCS observed particularly in the first year after AD diagnosis. Approximately 12.8% of prevalent adults in our study were defined as having moderate-to-severe disease based on dispensed treatments. This is relatively higher than the rate reported in another Israeli study by Shalom et al. [9], and lower than that estimated in another database study from Spain [18], which may be reflective of methodological differences in the treatment-based definitions.

Several methodological limitations should be noted. Our definition of AD may have missed some recently diagnosed patients, particularly in childhood (e.g., patients with only one pediatrician diagnosis, who did not yet have time to acquire additional diagnoses in their EMR). The study definition may also have misclassified some patients who did not have AD as having AD, due to limitations in the validity of ICD-9 code. Previous validation studies of AD algorithms have highlighted the trade-off between sensitivity and specificity in database studies [23]. Nonetheless, our results are in line with other database studies [10], and this limitation has been discussed in previous studies in MHS [8]. Data on AD disease severity were not available, and our definition of moderate-to-severe AD based on recent use of SI, biologics, and/or phototherapy may be subject to misclassification and did not distinguish between moderate and severe disease. In addition, as data on treatment indications were not available, some prior treatments (e.g., topical or systemic corticosteroids) may have been prescribed for indications other than AD. Nonetheless, though systemic corticosteroids are not generally recommended for AD [22], they are still frequently prescribed to treat moderate-to-severe AD [8]. A breakdown of specific treatments within the SI/biologic group was not available for this analysis. An analysis of treatment duration and discontinuation was beyond the scope of this descriptive study of trends over time. Data on long-term complications of AD treatment were not available for this study.

Taken together, we conclude that the incidence and prevalence rates of AD were comparable to those in similar database studies and remained relatively stable over the past decade. The results underscore the burden of medication use among children and adults, particularly in the first year after AD diagnosis, and the low rate of AD diagnosis among patients originally diagnosed as children 10 years earlier.

## Figures and Tables

**Table 1:** Characteristics of AD patients newly diagnosed in 2012–2021, by age group at AD diagnosis.

Characteristics at AD Diagnosis		Age at AD Diagnosis, Years			
		<12	12–17	18+	Total
Total		82,013 (100.0)	5672 (100.0)	36,865 (100.0)	124,550 (100.0)
Age, years	Median (IQR)	1.7 (0.6–4.2)	14.5 (13.2–16.2)	41.7 (30.0–55.6)	4.4 (1.1–26.0)
Sex	Male	43,663 (53.2)	2433 (42.9)	14,327 (38.9)	60,423 (48.5)
	Female	38,350 (46.8)	3239 (57.1)	22,538 (61.1)	64,127 (51.5)
Residence area	North	11,610 (14.2)	960 (16.9)	7402 (20.1)	19,972 (16.0)
	Sharon	18,838 (23.0)	1293 (22.8)	7213 (19.6)	27,344 (22.0)
	South	13,956 (17.0)	861 (15.2)	5279 (14.3)	20,096 (16.1)
	Center	18,376 (22.4)	1119 (19.7)	8347 (22.6)	27,842 (22.4)
	J-lem and Shfela	19,233 (23.5)	1439 (25.4)	8624 (23.4)	29,296 (23.5)
SES	Low	15,912 (19.4)	1019 (18.0)	5746 (15.6)	22,677 (18.2)
	Medium	22,470 (27.4)	1562 (27.5)	11,200 (30.4)	35,232 (28.3)
	High	43,557 (53.1)	3088 (54.4)	19,879 (53.9)	66,524 (53.4)
	Missing	73 (0.1)	3 (0.1)	40 (0.1)	116 (0.1)
BMI, kg/m^2 a^	<18.5	37,863 (46.2)	1824 (32.2)	1154 (3.1)	40,841 (32.8)
	18.5–24.9	4799 (5.9)	2386 (42.1)	14,384 (39.0)	21,569 (17.3)
	25.0–29.9	190 (0.2)	486 (8.6)	10,146 (27.5)	10,822 (8.7)
	≥30	85 (0.1)	172 (3.0)	6329 (17.2)	6586 (5.3)
	Missing	39,076 (47.6)	804 (14.2)	4852 (13.2)	44,732 (35.9)
Asthma	Ever	9540 (11.6)	1614 (28.5)	6909 (18.7)	18,063 (14.5)
	Prior 5 years	8162 (10.0)	508 (9.0)	3329 (9.0)	11,999 (9.6)
Allergic rhinitis	Ever	3235 (3.9)	1319 (23.3)	12,385 (33.6)	16,939 (13.6)
	Prior 5 years	3087 (3.8)	927 (16.3)	6536 (17.7)	10,550 (8.5)
Nasal polyposis	Ever	79 (0.1)	26 (0.5)	667 (1.8)	772 (0.6)
Ophthalmic conditions	Ever	11 (0.0)	9 (0.2)	8 (0.0)	28 (0.0)
Anemia—iron deficiency ^b^	Fe, tested	2763	828	10,506	14,097
	Low Fe (% in tested)	457 (16.5)	78 (9.4)	2602 (24.8)	3137 (22.3)
	Low Fe (% in total)	457 (0.6)	78 (1.4)	2602 (7.1)	3137 (2.5)
Anemia—low Hb ^b^	Hb, tested	33,209	2187	25,497	60,893
	Low Hb (% in tested)	4491 (13.5)	263 (12.0)	5607 (22.0)	10,361 (17.0)
	Low Hb (% in total)	4491 (5.5)	263 (4.6)	5607 (15.2)	10,361 (8.3)
Anemia ^b^	Low Hb/Fe (% in total)	4851 (5.9)	317 (5.6)	6957 (18.9)	12,125 (9.7)
Other comorbidities (ever)	Diabetes	15 (0.0)	15 (0.3)	2805 (7.6)	2835 (2.3)
	Hypertension	8 (0.0)	9 (0.2)	6665 (18.1)	6682 (5.4)
	CVD	881 (1.1)	63 (1.1)	2791 (7.6)	3735 (3.0)
	ADHD ^c^	956 (1.2)	1061 (18.7)	2467 (6.7)	4484 (3.6)
	Depression ^d^	38 (0.0)	148 (2.6)	7156 (19.4)	7342 (5.9)

^a^. Most recent measurement in the past 5 years; ^b^. most recent test in the past year; ^c^. diagnosed and treated; ^d^. depression and/or anxiety defined as diagnosed and treated, or undiagnosed with ≥2 dispensed prescriptions.

## Data Availability

All data generated or analyzed during this study are included in this published article and its Supplementary Information Files.

## Supplementary Materials

The following supporting information can be downloaded at: https://www.mdpi.com/article/10.3390/jcm13010281/s1, Figure S1: Selection of the AD prevalence population (2021), Figure S2: Selection of AD incidence cohort (2012–2021), Table S1: Characteristics of the AD prevalence population in 2021, by estimated disease severity, Table S2: Long-term patterns of AD diagnosis and treatment after 10 years.
