# Supplementary material for: Trends of Diagnosis, Disease Course, and Treatment of Atopic Dermatitis 2012–2021: Real-World Data from a Large Healthcare Provider
Source: J Clin Med. 2024 Jan 4;13(1):281. doi: 10.3390/jcm13010281 (PMC10779695; doi:10.3390/jcm13010281)
Supplement: Supplementary file 1 [file jcm-13-00281-s001.zip › jcm-2761210-supplementary.pdf]

Journal of
Clinical Medicine

Supplementary material

Contents

- Figure S1. Selection of the AD prevalence population (2021)
- **Figure S2.** Selection of AD incidence cohort (2012-2021)
- Table S1. Characteristics of the AD prevalence population in 2021, by estimated disease severity
- **Table S2.** Long-term patterns of AD diagnosis and treatment after 10 years

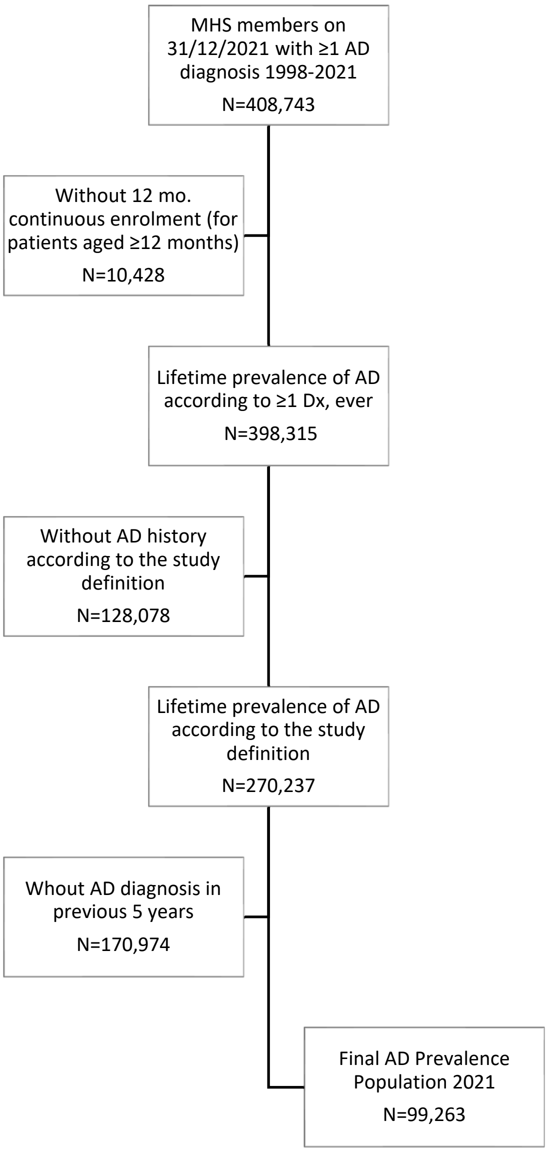

Figure S1. Selection of the AD prevalence population (2021)

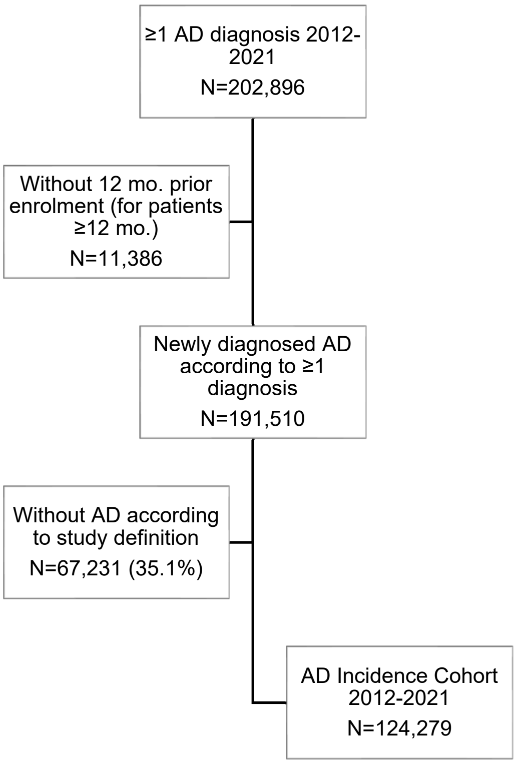

Figure S2. Selection of AD incidence cohort (2012-2021)

Journal of *Clinical Medicine*

MDPI

Characteristics of prevalent AD pateints on 31/12/2021		Mild AD				Moderate/severe AD			
		Age group, years				Age group, years			
		<12	12-17	18+	Total	<12	12-17	18+	Total
Total		44192 (100.0)	13375 (100.0)	32206 (100.0)	89773 (100.0)	4390 (100.0)	553 (100.0)	4721 (100.0)	9664 (100.0)
Age, years	Median (IQR)	6.2 (4.3-8.6)	14.5 (13.2-16.1)	37.1 (26.9-52.1)	12.2 (6.3-28.6)	4.4 (3.0-6.3)	14.7 (13.5-16.4)	48.9 (32.5-63.8)	16.8 (4.7-48.2)
Years since AD diagnosis	Median (IQR)	3.8 (2.2-5.5)	10.0 (4.2-13.0)	4.6 (2.2-16.0)	4.3 (2.4-8.7)	2.8 (1.7-4.6)	11.2 (5.0-13.7)	4.6 (1.9-15.8)	3.6 (1.8-8.1)
Sex	Male	22729 (51.4)	6386 (47.7)	11852 (36.8)	40967 (45.6)	2663 (60.7)	281 (50.8)	1746 (37.0)	4690 (48.5)
	Female	21463 (48.6)	6989 (52.3)	20354 (63.2)	48806 (54.4)	1727 (39.3)	272 (49.2)	2975 (63.0)	4974 (51.5)
Residence area	North	6189 (14.0)	1924 (14.4)	6357 (19.7)	14470 (16.1)	666 (15.2)	66 (11.9)	1005 (21.3)	1737 (18.0)
	Sharon	10291 (23.3)	3325 (24.9)	6354 (19.7)	19970 (22.2)	1040 (23.7)	146 (26.4)	932 (19.7)	2118 (21.9)
	South	7315 (16.6)	2111 (15.8)	4737 (14.7)	14163 (15.8)	804 (18.3)	97 (17.5)	791 (16.8)	1692 (17.5)
	Center	9836 (22.3)	2878 (21.5)	7198 (22.3)	19912 (22.2)	953 (21.7)	119 (21.5)	922 (19.5)	1994 (20.6)
	J-lem & Shfela	10561 (23.9)	3137 (23.5)	7560 (23.5)	21258 (23.7)	927 (21.1)	125 (22.6)	1071 (22.7)	2123 (22.0)
SES	Low	8053 (18.2)	1880 (14.1)	5044 (15.7)	14977 (16.7)	920 (21.0)	81 (14.6)	839 (17.8)	1840 (19.0)
	Medium	12431 (28.1)	3472 (26.0)	9555 (29.7)	25458 (28.4)	1274 (29.0)	127 (23.0)	1486 (31.5)	2887 (29.9)
	High	23662 (53.5)	8012 (59.9)	17571 (54.6)	49245 (54.9)	2192 (49.9)	344 (62.2)	2391 (50.6)	4927 (51.0)
	Missing	46 (0.1)	11 (0.1)	36 (0.1)	93 (0.1)	4 (0.1)	1 (0.2)	5 (0.1)	10 (0.1)
BMI, kg/m^2 [a]	<18.5	30005 (67.9)	4526 (33.8)	1145 (3.6)	35676 (39.7)	2841 (64.7)	148 (26.8)	130 (2.8)	3119 (32.3)
	18.5-24.9	4120 (9.3)	5482 (41.0)	12508 (38.8)	22110 (24.6)	398 (9.1)	266 (48.1)	1525 (32.3)	2189 (22.7)
	25.0-29.9	380 (0.9)	1133 (8.5)	7837 (24.3)	9350 (10.4)	27 (0.6)	68 (12.3)	1481 (31.4)	1576 (16.3)
	≥30	63 (0.1)	413 (3.1)	5056 (15.7)	5532 (6.2)	11 (0.3)	18 (3.3)	1121 (23.7)	1150 (11.9)
	Missing	9624 (21.8)	1821 (13.6)	5660 (17.6)	17105 (19.1)	1113 (25.4)	53 (9.6)	464 (9.8)	1630 (16.9)
Asthma	Ever	8380 (19.0)	4427 (33.1)	8682 (27.0)	21489 (23.9)	1730 (39.4)	304 (55.0)	1759 (37.3)	3793 (39.2)
	Prior 5y	5730 (13.0)	1416 (10.6)	3282 (10.2)	10428 (11.6)	1616 (36.8)	187 (33.8)	1028 (21.8)	2831 (29.3)
Allergic rhinitis	Ever	4874 (11.0)	3923 (29.3)	13775 (42.8)	22572 (25.1)	656 (14.9)	252 (45.6)	2447 (51.8)	3355 (34.7)
	Prior 5y	4024 (9.1)	2480 (18.5)	6376 (19.8)	12880 (14.3)	597 (13.6)	172 (31.1)	1238 (26.2)	2007 (20.8)
Nasal polyposis	Ever	121 (0.3)	62 (0.5)	614 (1.9)	797 (0.9)	11 (0.3)	7 (1.3)	196 (4.2)	214 (2.2)
Ophthalmic conditions	Ever	24 (0.1)	28 (0.2)	21 (0.1)	73 (0.1)	2 (0.0)	0 (0.0)	8 (0.2)	10 (0.1)
Anemia – iron deficiency [b]	Fe, tested	3175	2133	10774	16082	395	126	2064	2585
	Low Fe (% in tested)	38 (1.2)	28 (1.3)	2365 (22.0)	2431 (15.1)	13 (3.3)	3 (2.4)	542 (26.3)	558 (21.6)
	Low Fe (% in total)	38 (0.1)	28 (0.2)	2365 (7.3)	2431 (2.7)	13 (0.3)	3 (0.5)	542 (11.5)	558 (5.8)
Anemia – low Hb [b]	Hb, tested	12951	5171	21066	39188	1938	304	3817	6059

	Low Hb (% in tested)	1425 (11.0)	557 (10.8)	4944 (23.5)	6926 (17.7)	266 (13.7)	34 (11.2)	912 (23.9)	1212 (20.0)
	Low Hb (% in total)	1425 (3.2)	557 (4.2)	4944 (15.4)	6926 (7.7)	266 (6.1)	34 (6.1)	912 (19.3)	1212 (12.5)
Anemia [b]	Low Hb/Fe (% in total)	1449 (3.3)	571 (4.3)	6233 (19.4)	8253 (9.2)	270 (6.2)	35 (6.3)	1211 (25.7)	1516 (15.7)
	Diabetes	30 (0.1)	38 (0.3)	1973 (6.1)	2041 (2.3)	2 (0.0)	0 (0.0)	511 (10.8)	513 (5.3)
	Hypertension	12 (0.0)	22 (0.2)	4490 (13.9)	4524 (5.0)	3 (0.1)	1 (0.2)	1266 (26.8)	1270 (13.1)
Other comorbidities (ever)	CVD	576 (1.3)	212 (1.6)	2043 (6.3)	2831 (3.2)	82 (1.9)	16 (2.9)	559 (11.8)	657 (6.8)
	ADHD [c]	1643 (3.7)	2875 (21.5)	4432 (13.8)	8950 (10.0)	90 (2.1)	136 (24.6)	559 (11.8)	785 (8.1)
	Depression [d]	101 (0.2)	498 (3.7)	6944 (21.6)	7543 (8.4)	5 (0.1)	32 (5.8)	1569 (33.2)	1606 (16.6)

[a.] Most recent measurement in the past 5 years; [b.] Most recent test in the past year; [c.] diagnosed and treated; [d.] depression and/or anxiety defined as diagnosed and treated, or undiagnosed with ≥2 dispensed prescriptions.

Table S1. Characteristics of the AD prevalence population in 2021, by estimated disease severity

Journal of
Clinical Medicine

MDPI

AD patients newly diagnosed in 2012		Age at diagnosis, years		
		<18 (N=7155)	18+ (N=3949)	Total (N=11104)
≥1 AD diagnosis		6.1%	3.8%	5.3%
≥1 dispensed AD treatment,	Any below	19.1%	35.9%	25.1%
	Any TCS	18.2%	34.7%	24.1%
	TCI	1.9%	2.3%	2.0%
	PDE4i	0.4%	0.7%	0.5%
	Phototherapy	0.2%	0.6%	0.3%
	SI/Biologic	0.4%	1.3%	0.7%
≥1 AD diagnosis and/or treatment		20.6%	36.2%	26.1%

AD patients newly diagnosed and treated in 2012		Age at diagnosis, years		
		<18 (N=5248)	18+ (N=3223)	Total (N=8471)
≥1 AD diagnosis		6.4%	3.9%	5.5%
≥1 dispensed AD treatment,	Any below	20.1%	38.0%	26.9%
	Any TCS	19.2%	36.7%	25.9%
	TCI	2.1%	2.5%	2.2%
	PDE4i	0.4%	0.8%	0.6%
	Phototherapy	0.2%	0.7%	0.4%
	SI/Biologic	0.5%	1.5%	0.9%
≥1 AD diagnosis and/or treatment		21.5%	38.3%	27.9%

Table S2. Long-term patterns of AD diagnosis and treatment after 10 years
